# Investigating the specificity of the neurologic pain signature against breathlessness and finger opposition

**DOI:** 10.1097/j.pain.0000000000002327

**Published:** 2021-12-01

**Authors:** Olivia K. Harrison, Anja Hayen, Tor D. Wager, Kyle T.S. Pattinson

**Affiliations:** a Translational Neuromodeling Unit, Institute of Biomedical Engineering, University of Zurich and ETH Zurich, Zurich, Switzerland; b School of Pharmacy, University of Otago, New Zealand; c Nuffield Department of Clinical Neurosciences, University of Oxford, Oxford, United Kingdom; d Wellcome Centre for NeuroImaging, University of Oxford, Oxford, United Kingdom; e School of Psychology & Clinical Language Sciences, University of Reading, Reading, United Kingdom; f USA Department of Psychological and Brain Sciences, Dartmouth College, Hanover, United States.

**Keywords:** Pain, Breathlessness, Interoception, Threat

## Abstract

Brain biomarkers of pain, including pain-predictive “signatures” based on brain activity, can provide measures of neurophysiological processes and potential targets for interventions. A central issue relates to the specificity of such measures, and understanding their current limits will both advance their development and explore potentially generalizable properties of pain to other states. Here, we used 2 data sets to test the neurologic pain signature (NPS), an established pain neuromarker. In study 1, brain activity was measured using high-field functional magnetic resonance imaging (7T fMRI, N = 40) during 5 to 25 seconds of experimental breathlessness (induced by inspiratory resistive loading), conditioned breathlessness anticipation, and finger opposition. In study 2, we assessed anticipation and breathlessness perception (3T, N = 19) under blinded saline (placebo) and remifentanil administration. The NPS responded to breathlessness, anticipation, and finger opposition, although no direct comparisons with painful events were possible. Local NPS patterns in anterior or midinsula, S2, and dorsal anterior cingulate responded to breathlessness and finger opposition and were reduced by remifentanil. Local NPS responses in the dorsal posterior insula did not respond to any manipulations. Therefore, significant global NPS activity alone is not specific for pain, and we offer insight into the overlap between NPS responses, breathlessness, and somatomotor demand.

## 1. Introduction

Although perceptions of pain are often identified and assessed through subjective self-report, the experience of pain can be influenced by higher cognitive functions such as attention^84^ and expectation.^2^ Therefore, the quest has begun for biological “readouts” related to pain in the brain, with the hope of allowing us to assess pain-related neurophysiology within individuals using noninvasive neuroimaging measures.^83,88^ Brain “neuromarkers” or “signatures” could provide biological measures for characterizing subtypes of pain or individual function and pathology^11,22,80^ and neurophysiological targets for treatments. They could also augment pain detection and characterization when self-report measures are unavailable or otherwise problematic.^24^ These tools are designed to identify pain across experiments and laboratories and eventually lead to use in those who cannot accurately express pain for themselves.

Here, we focus on the neurologic pain signature (NPS), a statistical model of the distributed pattern of activity across brain regions associated with pain. The NPS uses functional magnetic resonance imaging (fMRI) signals in major targets of nociceptive pathways (dorsal posterior insula, ventrolateral and medial thalamus, insula, anterior midcingulate, amgydala, periaqueductal gray, and hypothalamus), which contribute to increased predicted pain when active, along with some other regions that are not clearly nociceptive. Applying the NPS entails calculating a weighted average across voxels in a functional brain image from a given test participant and can be applied to fMRI images from new individuals and cohorts. Local pattern weights limited to individual anatomical regions can also be used to obtain local pattern responses.^88^ The NPS has been tested on more than 40 unique participant cohorts to date (for reviews, see Refs. 45,87); this approach to evaluating generalizability and specificity across diverse cohorts is part of a trend in neuroimaging research using pattern information to assess pain and other cognitive and affective processes^60,62,63,70,81^ and develop robust and useful fMRI measures.^86^

The NPS has demonstrated specificity to pain in previous studies: The model does not respond appreciably to the brain activity evoked by nonnoxious warm stimuli,^83^ threat cues,^46,58,83^ social rejection-related stimuli,^83^ observed pain,^46^ or aversive images,^16^ among others, and brain-wide activity patterns related to these states are dissociable from those elicited during somatic pain. Evidence to date suggests the NPS tracks pain of primarily nociceptive origin (including thermal, mechanical, laser, visceral, and electrical^46,56,91,93^), whereas it does not respond to social “pain”.^46,88^ It is not strongly influenced by most forms of placebo treatment,^93^ cognitive regulation,^89^ reward,^8^ knowledge about drug-delivery context,^83,93^ or perceived control.^12^ On the other hand, the NPS shows significant responses to remifentanil,^83,93^ citalopram (in some individuals),^58^ and some types of psychosocial or behavioral manipulations,^42,44^ showing promise as a pharmacodynamic biomarker. These findings underscore the idea that the NPS and other brain measures do not “measure pain” (a subjective experience), but rather measure specific neurophysiological processes linked to pain construction.

However, the NPS has not yet been tested against predominantly somatosensory aversive stimuli. One ideal test case might be the frightening perception of breathlessness; a multidimensional symptom that induces fear and suffering across a broad range of individuals.^34,38,61^ Although the definition of breathlessness (or “dyspnea”, “a subjective experience of breathing discomfort that consists of qualitatively distinct sensations that vary in intensity”) from the American Thoracic Society^3^ closely parallels that of pain,^67^ clinical experiences of breathlessness have been described to include qualities such as “air hunger,” “chest tightness,” and “work of breathing.”^4–6,57–59,67,75^ Here we use the term “breathlessness” as an equivalent to “dyspnea,” recognising that it more closely reflects the language used by patients, although more recent work has come to recognise that lived experienced of both “dyspnea” and “breathlessness” are subjective, highly varied and specific to each individual.^34,38,59^ In this study, we used brief inspiratory resistive loading as an experimental model to induce breathlessness in our volunteers. We additionally used a delay-conditioning paradigm to model some of the learned anticipatory fears that patients encounter.^61^ Although the perceptions of inspiratory loading are biased towards the “work-effort” dimension, the stimulus provides an experimental model that is convenient for fMRI experiments and thus is valuable for informing translational research. Here, inspiratory resistive loads were applied to measure the brain activity associated with increased breathing effort, and previous work has noted many similarities between brain networks associated with experimentally induced breathlessness and pain.^5,25,51–53,55,68,71,82^

We aimed to test the specificity of the NPS^83^ using 2 data sets that induced both the anticipation and perception of breathlessness (induced by inspiratory resistance; study 1^27^ and study 2^35^). In addition, study 1 included a simple somatomotor task of finger opposition, and study 2 included tasks performed after infusions of the opioid remifentanil or saline (placebo). We aimed to understand existing limitations and generalizable properties of the NPS, support its refinement towards greater pain specificity, and investigate the local patterns of brain activity that could be shared between the NPS and salient somatomotor stimuli such as breathlessness.

## 2. Methods

### 2.1. Study 1

#### 2.1.1. Participants

Forty healthy, right-handed individuals were recruited (20 males, 20 females; mean age ± SD, 26 ± 7 years) as part of wider healthy volunteer study,^27^ with no history of smoking or neurological disease. Half of the participants were recruited because they regularly participated in endurance sport, and half were age-matched and sex-matched sedentary participants. Any group differences were not considered in this analysis. Participants completed a conditioning session and one fMRI session on 2 consecutive days.

#### 2.1.2. Magnetic resonance imaging scanning sequences

Data were acquired with a 7T Siemens Magnetom scanner, with 70 mT/m gradient strength and a 32-channel Rx, single-channel birdcage Tx head coil (Nova Medical, MA). A T2*-weighted gradient echo planar image (EPI) was used for functional scanning (sequence parameters: echo time [TE], 24 ms; repetition time [TR], 3 seconds; voxel size, 2 × 2 × 2 mm; number of slices, 63; field of view [FOV], 220 mm; number of volumes, 550). A T1-weighted structural scan (MP-RAGE: magnetisation-prepared rapid gradient-echo, sequence parameters: TE, 2.96 ms; TR, 2200 ms; voxel size, 0.7 × 0.7 × 0.7 mm) and a fieldmap (matched to EPI FOV) were also acquired for registration of functional images.

#### 2.1.3. Stimuli and tasks

Participants were trained using an aversive delay-conditioning paradigm to associate a simple shape with an upcoming inspiratory resistance stimulus (approximately −15 cm H_2_O; 100% contingency pairing) and a second shape with no upcoming inspiratory resistance (0% contingency pairing with inspiratory resistance). The resistance-related symbol was presented on the screen for 30 seconds, which included a varying 5 to 15 seconds anticipation period before the loading was applied. The unloaded breathing symbol was presented for 20 seconds, and each condition was repeated 14 times in a semirandomised order. A finger opposition task was also included in the protocol, where the word “TAP” was presented for 15 seconds on the screen (10 repeats), and participants were asked to perform an opposition movement between their right thumb and fingers. After every stimulus period, participants were asked to rate the difficulty of the previous stimulus using a visual analogue scale (VAS) with a sliding bar between “not at all difficult” and “extremely difficult.” Participants were also asked to rate how anxious each symbol made them feel using a VAS between “not at all anxious” (0%) and “extremely anxious” (100%) immediately after the functional magnetic resonance imaging protocol.

### 2.2. Study 2

#### 2.2.1. Participants

Nineteen healthy participants (9 males, 10 females; mean age ± SD, 24 ± 7 years) completed this double-blind, randomized, placebo-controlled study of the opioid remifentanil, with no history of smoking or neurological disease.^35^ See 35 for full information on excluded participants. Each participant completed a conditioning session and 2 fMRI sessions (remifentanil or saline placebo, counterbalanced order) on 3 consecutive days.

#### 2.2.2. Magnetic resonance imaging scanning sequences

Data were acquired with a 3T Siemens Trio scanner, with a 32-channel head coil. A T2*-weighted gradient EPI was used for functional scanning (sequence parameters: TE, 30 ms; TR, 3 seconds; voxel size, 3 × 3 × 3 mm; number of slices, 45; FOV, 192 mm; number of volumes, 380). A T1-weighted structural scan (MP-RAGE, sequence parameters: TE, 4.68 ms; TR, 1720 ms; voxel size, 1 × 1 × 1 mm) and a fieldmap (matched to EPI FOV) were also acquired for registration of functional images.

#### 2.2.3. Stimuli and tasks

Participants were trained using an aversive delay-conditioning paradigm to associate a simple shape with either a moderate (approximately −12 cm H_2_O) or mild (approximately −3 cm H_2_O) upcoming inspiratory resistance stimulus (100% contingency pairing; 4 repeats of each) and a different shape with no upcoming inspiratory resistance (0% contingency pairing with inspiratory resistance; 8 repeats). The resistance-related symbols were presented on the screen for 38 to 68 seconds, which included an 8-second anticipation period before the loading was applied. The unloaded breathing symbol was presented for 27 to 47 seconds. After every stimulus period, participants were asked to rate the intensity of the previous stimulus using a VAS with a sliding bar between “no breathlessness” and “severe breathlessness” and the unpleasantness between “not unpleasant” and “extremely unpleasant”. Participants were also asked to rate the Bond–Lader mood values of tension–relaxation, sedation–alertness, and discontentment–contentment^10^ immediately after the functional magnetic resonance imaging protocol. These scales were not used in the current analysis, and readers are referred to the original publication for further information.^35^ Mild resistance stimuli were not considered in the current analyses to remain consistent with data provided from study 1.

#### 2.2.4. Drug infusion

A double-blinded target-controlled infusion pump (Graseby 3500 target-controlled infusion incorporating Diprisor; SIMS Graseby Ltd, Watford, United Kingdom) delivered either remifentanil (10 μg/mL, with an effect site concentration of 0.7 ng/mL) or saline placebo through a cannula placed in the dorsum of the left hand for a total of 45 minutes, including a 10-minute ramp-up period to reach the desired effect site concentration. All participants fasted for 6 hours before each visit and were monitored for an hour after termination of the infusion.

### 2.3. Physiological measures

In both data sets, extensive physiological measures were taken to both measure the effects of the inspiratory resistance stimuli delivered and provide the data required for rigorous noise correction procedures. Chest movements were measured using respiratory bellows (nonmetallic pneumographic belt; Lafayette Instrument Company, Lafayette), and heart rate was measured using a pulse oximeter (9500 Multigas Monitor; MR Equipment Corp., NY). End-tidal pressure of carbon dioxide (P_ET_CO_2_) and oxygen (P_ET_O_2_) were sampled using a port beside the mouthpiece of the breathing system. Expired gases were determined using a rapidly responding gas analyser (ADInstruments Ltd, Oxford, United Kingdom) and pressure using a pressure transducer (MP 45, ± 50 cmH_2_O, Validyne Corp., Northridge, CA) connected to an amplifier (Pressure transducer indicator, PK Morgan Ltd, Kent, United Kingdom). All physiological measurement devices were connected to a data acquisition device (PowerLab; ADInstruments Ltd) coupled to a desktop computer with recording software (LabChart 7 in study 1 and LabChart 5 in study 2; ADInstruments Ltd). To minimise the confounds associated with increases in P_ET_CO_2_ induced by inspiratory resistive loading, small boluses of additional, repeated CO_2_ were interspersed during rest periods in study 1, and in study 2, end-tidal measurements were held constant by initially increasing P_ET_CO_2_ by 0.3 kPa and then manually adjusting inspired CO_2_ as necessary.

### 2.4. Data preprocessing

Image preprocessing was performed using FEAT (version 6, part of FSL: www.fmrib.ox.ac.uk/fsl), using a whole-brain approach. The preprocessing methods used were as follows: motion correction and motion parameter recording (MCFLIRT: Motion Correction using FMRIB’s Linear Image Registration Tool^41^), removal of the nonbrain structures (skull and surrounding tissue) (BET: Brain Extraction Tool^78^), spatial smoothing using a full-width half-maximum Gaussian kernel of 2 mm for study 1 and 5 mm for study 2 (adjusted for the different voxel sizes and scanner strength and in line with the original study results), and high-pass temporal filtering (Gaussian-weighted least-squares straight-line fitting) of 120 seconds for study 1 and 75 seconds for study 2. Registration from EPI to structural scans was performed using boundary-based registration (BBR^31^; 6 degrees of freedom) with fieldmap distortion correction and from structural to standard space was using an affine transformation followed by nonlinear registration (FNIRT: FMRIB’s Nonlinear Registration Tool^1^).

Data denoising was conducted using a combination of independent component analysis (ICA) and retrospective image correction (RETROICOR^13,33^), as previously described.^26,27,35^ This process involved decomposing the data using automatic dimensionality estimation.^43^ Head motion regressors calculated from the motion correction preprocessing step were regressed out of the data alongside the noise components identified during ICA denoising^32^ before the first-level fitting of the task-based general linear model. Physiological recordings of heart rate and respiration from respiratory bellows were transformed into cardiac, respiratory, and interaction harmonics, as well as a measure of respiratory volume per unit of time (RVT)^13,33^ corresponding to each acquisition slice, and the signal associated with this noise was isolated using linear regression, adjusted for any interaction with previously identified ICA noise components and then subtracted from the data.

### 2.5. General linear model

A general linear model was used to describe the data from each participant. Regressors were generated for anticipation periods for each level of loading (ie, one anticipation condition for study 1 and one mild and one moderate anticipation condition for study 2) from the beginning of the symbol presentation until the onset of the inspiratory resistive loading. Corresponding inspiratory resistance regressors were then constructed from the onset of the resistance stimulus until the end of the loading period. Unloaded periods were modelled for the duration of the unloaded stimulus, and study 1 also included a regressor covering the periods of finger opposition. Both immediate recovery and rating periods when participants rated each stimulus were modelled as regressors of no interest, and demeaned trial-by-trial ratings of each stimulus were included to model out any intertrial variability. P_ET_CO_2_ was entered as a separate regressor to account for fluctuations that could affect the BOLD signal.^15,86^ All regressors were convolved with an optimal basis set of 3 waveforms (FLOBS^92^) to account for possible changes in the haemodynamic response function and any slice-timing delays. The second and third FLOBS waveforms—which model approximations to the temporal and dispersion derivatives—were orthogonalised to the first waveform, of which the parameter estimate was then passed up to the higher level to be used in both univariate and NPS group analyses.

The contrasts of interest that were analysed for study 1 were anticipation > no resistance cue (“anticipation” contrast), resistance > no resistance (“breathlessness” contrast), and finger opposition > baseline (“finger opposition” contrast). The contrasts of interest that were analysed for study 2 were anticipation of resistance > no resistance cue in the saline condition (“anticipation” contrast), resistance > no resistance in the saline condition (“breathlessness” contrast), anticipation of resistance > no resistance cue in the remifentanil condition (“remi anticipation” contrast), and resistance > no resistance in the remifentanil condition (remi breathlessness contrast). The difference between saline and remifentanil conditions was also compared for both anticipation and breathlessness contrasts.

### 2.6. Univariate analyses

The mean group activity for each contrast of interest in both data sets was calculated using nonparametric analyses using FSL’s randomize tool,^85^ cluster corrected with T > 2.3 and visualised using a threshold of *P* < 0.5 family-wise error-corrected results. This threshold was chosen to display the pattern of activity rather than only the activity that would survive rigorous significance testing at *P* < 0.05.

### 2.7. Neurologic pain signature analyses

For each contrast in each study, we calculated the overall NPS response as specified by Wager et al.^83^ This entailed taking the dot product of the NPS weight map and each test contrast image from each individual participant and calculating a weighted average over each test image, where the NPS map specifies the weights. It reduces each contrast image to a single number, the “NPS response,” which is the predicted pain intensity based on the model. We tested whether the NPS responses were significantly different from zero using two-sided Student *t* tests. This is mathematically equivalent to conducting paired *t* tests on within-person contrasts, treating participant as a random effect. We also applied the local NPS patterns from nociceptive target regions with predominantly positive weights (“NPS positive” subregions) and regions with negative weights (“NPS negative” subregions), as defined in Refs. 46, 57. We use a standard threshold of *P* < 0.05 for statistical significance in these a priori tests, and also note tests that are significant at *P* < 0.01 and q < 0.05, a false discovery rate corrected for the number of contrasts considered within each data set.

## 3. Results

### 3.1. Behavioural results

The main physiological and self-report results for each of the data sets are presented in Tables 1 and 2. Inspiratory loading achieved moderate increases in inspiratory pressure in both studies (Table 1), alongside moderate self-report ratings of intensity and unpleasantness, with mild reports of anxiety (Table 2).

### 3.2. Global neurologic pain signature results

**Figure 1.**
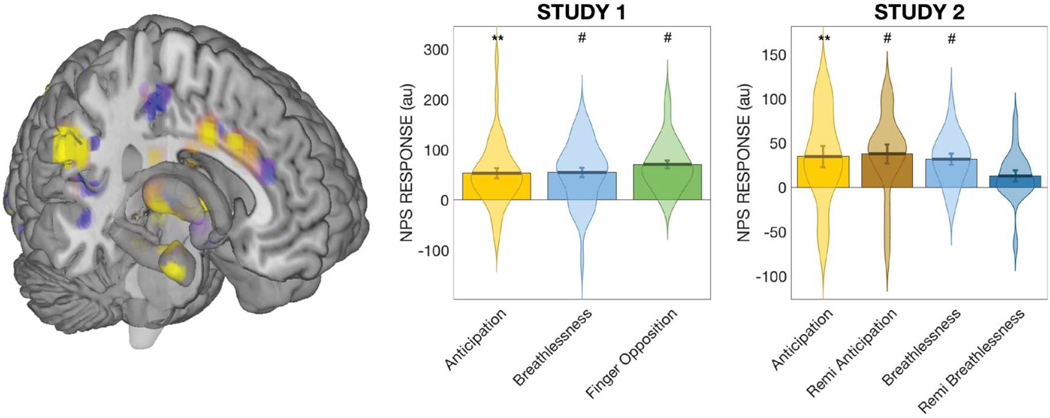
Overall NPS activity in the contrasts of interest for the 2 data sets. Left: Three-dimensional representation of some of the core regions of the NPS. **Significantly different from zero at *P* < 0.01; #Significantly different from zero at q < 0.05 (FDR corrected). NPS, neurologic pain signature.

Anticipation of inspiratory resistance, resistance perception, and finger opposition all significantly activated the overall NPS (Table 1 and Fig. 1), and the findings for anticipation and inspiratory resistance were replicated across both independent data sets. The administration of remifentanil in study 2 did not alter the NPS response to anticipation of resistance, and although it seemed to reduce the response to inspiratory resistance itself, this did not reach statistical significance (Table 3).

### 3.3. Univariate results

**Figure 2.**
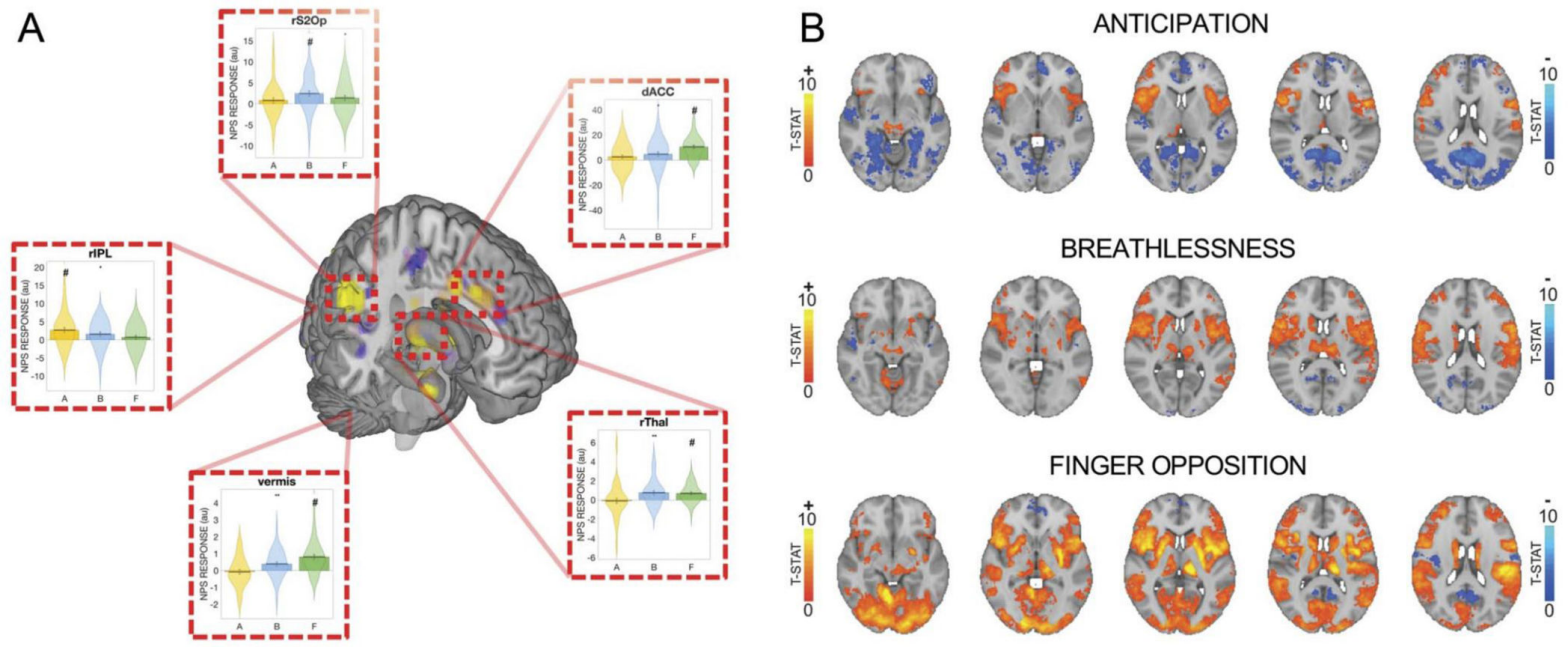
(A) Regional NPS activity subregions of the NPS for the anticipation, breathlessness, and finger opposition contrasts from study 1. Significant NPS activation is observed in the dorsal anterior cingulate cortex (dACC), right thalamus (rThal), right secondary somatosensory cortex or operculum (rS2Op), and vermis for both breathlessness and finger opposition and in the right inferior parietal lobule (rIPL) for both anticipation and breathlessness. For a full list of regions please see Supplementary Table 1B, available at http://links.lww.com/PAIN/B381. (B) Univariate statistical maps (displayed using a visualisation threshold of *P* < 0.5) created using voxel-wise permutation testing with a cluster-forming threshold of T > 2.3 for each of the contrasts of interest. A, anticipation contrast; B, breathlessness contrast; F, finger opposition contrast. *Significantly different from zero at *P* < 0.05; **Significantly different from zero at *P* < 0.01; #Significantly different from zero at q < 0.05 (FDR corrected). NPS, neurologic pain signature.

**Figure 3.**
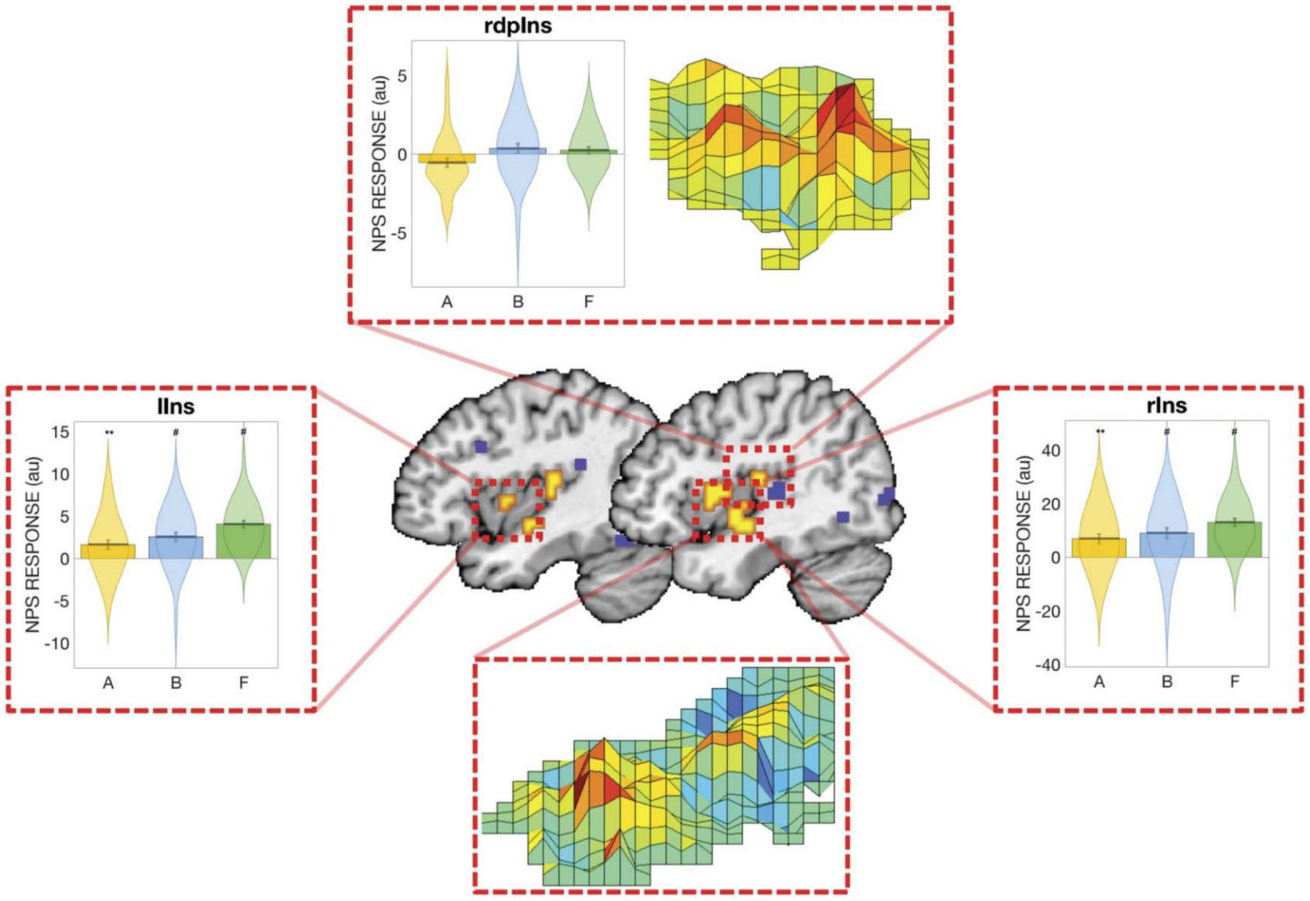
Regional NPS activity in the insula for the anticipation, breathlessness, and finger opposition contrasts from study 1. Robust statistical activity is observed in the bilateral insula (labelled lIns and rIns) for all 3 conditions, whereas no significant activity is observed in the right dorsal posterior insula (rdpIns). A, anticipation contrast; B, breathlessness contrast; F, finger opposition contrast. **Significantly different from zero at *P* < 0.01; #Significantly different from zero at q < 0.05 (FDR corrected). NPS, neurologic pain signature.

Results of the univariate analyses are presented in Figures 2 and 3, providing maps of the voxel-wise activity of each contrast of interest.

### 3.4. Study 1 regional neurologic pain signature results

Within the NPS subregions, the anticipation contrast produced significant responses in the “positive NPS” regions (whose weights were largely positive, increasing predicted pain) of the bilateral anterior or midinsula, and significant responses in the “negative NPS” regions (regions with largely negative weights) of the bilateral lateral occipital cortex and right inferior parietal lobule (Figs. 2 and 3; Supplementary Table 1, available at http://links.lww.com/PAIN/B381). During inspiratory resistance, significant responses were observed in the positive NPS regions of the bilateral insula, right thalamus, right secondary sensory cortex, dorsal anterior cingulate cortex (dACC), and vermis and significant responses in the negative NPS region of the right inferior parietal lobule (Figs. 2 and 3; Supplementary Table 1, available at http://links.lww.com/PAIN/B381). Consistent with the breathlessness contrast, finger opposition also produced significant responses in the positive NPS regions of the bilateral insula, right thalamus, right secondary sensory cortex, dACC, and vermis, plus additional activity in the right primary visual cortex. In the negative NPS regions, finger opposition activated the lateral occipital cortex and right posterior lateral occipital cortex (Supplementary Table 1, available at http://links.lww.com/PAIN/B381). No contrasts produced significant activity in the right dorsal posterior insula subregion of the NPS (Fig. 2). Full statistical reports and visualisations of the raw condition-related activity are provided in the supplementary material (available at http://links.lww.com/PAIN/B381).

### 3.5. Study 2 regional neurologic pain signature results

**Figure 4.**
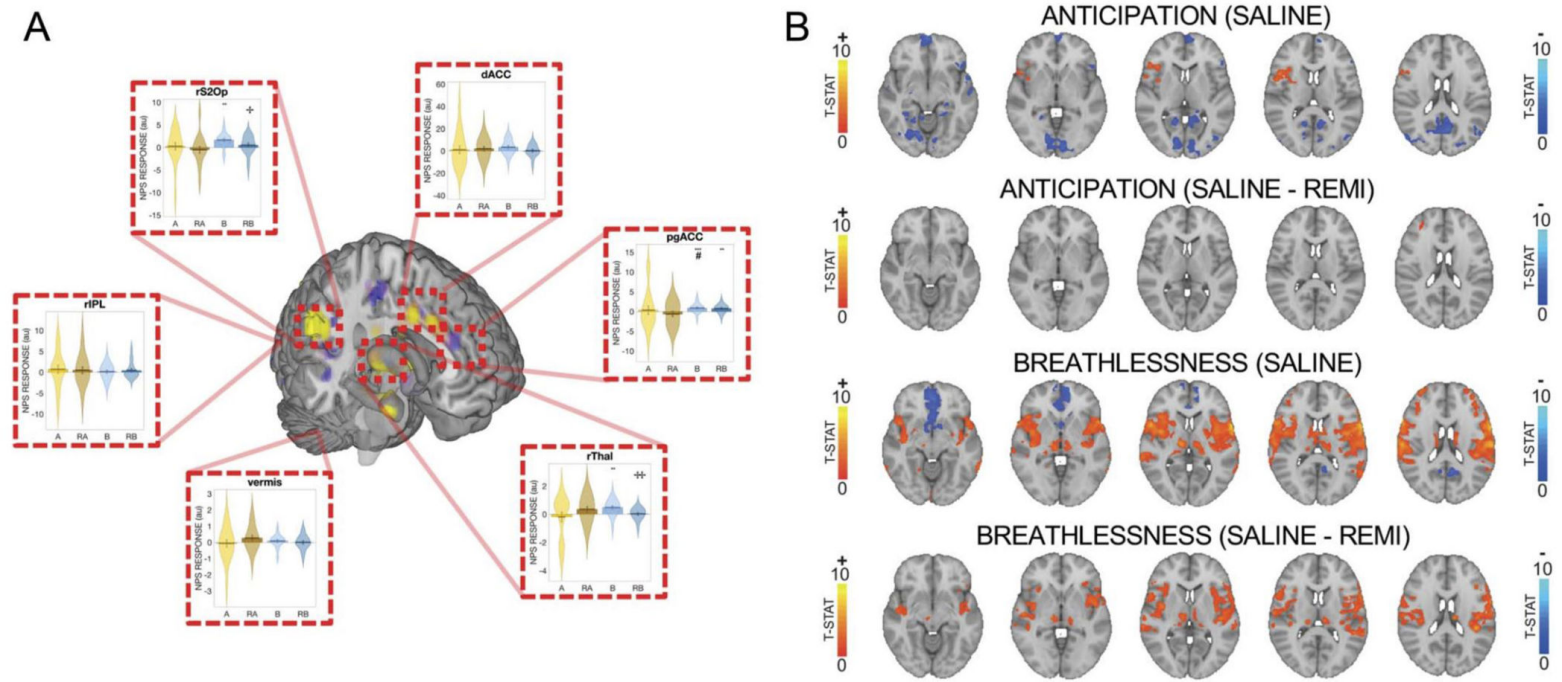
(A) Regional NPS activity subregions of the NPS for the anticipation and breathlessness contrasts during both saline and remifentanil administration from study 2. Significant NPS activation is observed in the dorsal and pregenual anterior cingulate cortex (dACC and pgACC), right thalamus (rThal), and right secondary somatosensory cortex or operculum (rS2Op) for breathlessness, with the NPS-related activity in the right thalamus and rS2Op significantly modulated by the administration of the opioid remifentanil. For a full list of regions please see Supplementary Table 2B, available at http://links.lww.com/PAIN/B381. (B) Univariate statistical maps (displayed using a visualisation threshold of *P*< 0.5) created using voxel-wise permutation testing with a cluster-forming threshold of T > 2.3 for each of the contrasts of interest. A, anticipation contrast (saline); RA, remifentanil anticipation contrast; B, breathlessness contrast (saline); RB, remifentanil breathlessness contrast. *Significantly different from zero at *P* < 0.05; **Significantly different from zero at *P* < 0.01; #Significantly different from zero at q < 0.05 (FDR corrected); ^✢^Significantly modulated by remifentanil with *P* < 0.05; ^✢✢^Significantly modulated by remifentanil at *P* < 0.01. dACC, dorsal anterior cingulate cortex; NPS, neurologic pain signature.

**Figure 5.**
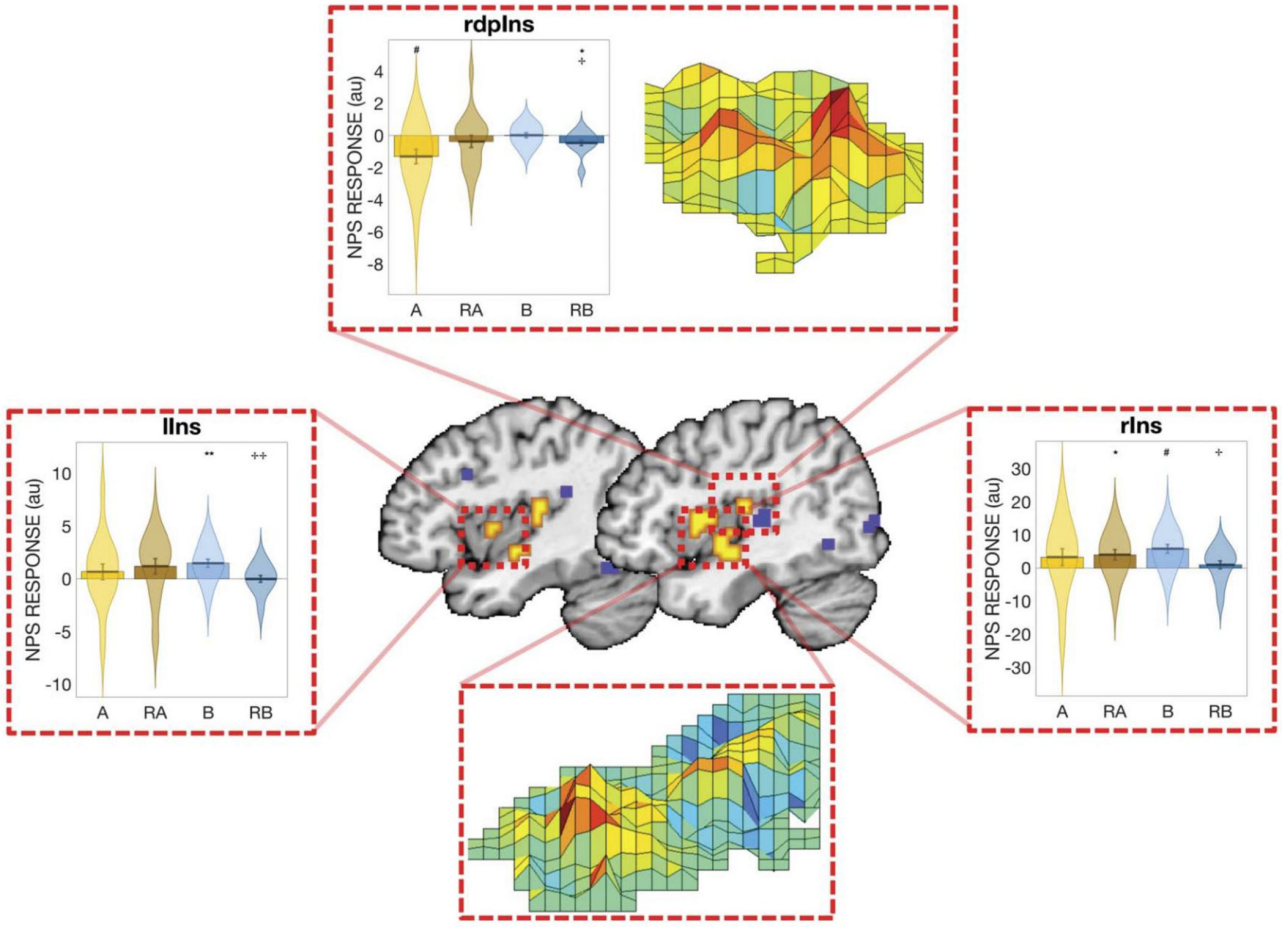
Regional NPS activity in the insula for the anticipation and breathlessness contrasts during both saline and remifentanil administration from study 2. Robust, positive statistically significant NPS-related activity is only observed in the bilateral insula (labelled lIns and rIns) for the breathlessness contrast, which is significantly modulated by the administration of the opioid remifentanil. Neurologic pain signature–related activity in the right dorsal posterior insula (rdpIns) is significantly decreased during saline anticipation. A, anticipation contrast (saline); RA, remifentanil anticipation contrast; B, breathlessness contrast (saline); RB, remifentanil breathlessness contrast. **Significantly different from zero at *P* < 0.01; #Significantly different from zero at q < 0.05 (FDR corrected); ^✢✢^Significantly modulated by remifentanil at *P* < 0.05.

Within the positive NPS subregions in study 2, the anticipation (saline) contrast produced a significant response in the right primary visual cortex, with a negative response in the right dorsal posterior insula (Figs. 4 and 5; Supplementary Table 2, available at http://links.lww.com/PAIN/B381). No significant responses were found in the negative NPS subregions. The administration of remifentanil did not significantly modulate any of the NPS-related subregion activity during anticipation, although the right anterior or midinsula (positive region) and right posterior lateral occipital cortex and left superior temporal sulcus (negative regions) all additionally produced significant results (Figs. 4 and 5; Supplementary Table 2, available at http://links.lww.com/PAIN/B381).

During inspiratory resistance, the positive NPS regions of bilateral anterior or midinsula, right thalamus, right secondary sensory cortex, and dACC produced significant NPS-related activity, whereas the negative NPS subregion of the pregenual anterior cingulate cortex was also significant (Figs. 4 and 5; Supplementary Table 2, available at http://links.lww.com/PAIN/B381). The administration of remifentanil significantly decreased the NPS-related activity in all saline significant regions except the pregenual anterior cingulate cortex and additionally produced a significant decrease in the right dorsal posterior insula (Figs. 4 and 5; Supplementary Table 2, available at http://links.lww.com/PAIN/B381).

## 4. Discussion

### 4.1. Main findings

Using 2 independent data sets, we have demonstrated that both the anticipation and experience of experimental breathlessness robustly evoked significant activity in an established pain signature (the NPS^83^). Neurologic pain signature-related activity during this breathlessness was reduced by the short-acting opioid remifentanil (study 2). Furthermore, a somatomotor finger opposition task was also able to evoke significant responses in the NPS and several constituent subregions, including anterior or midinsula, thalamus, and secondary somatosensory cortex (S2). The activity in these areas may thus provide a general substrate for common somatomotor activity and related processes—for example, action policy selection and execution—that underlie responses to pain and other challenges.

These results are somewhat surprising because the NPS has not responded in previous studies to multiple salient, affective challenges^60,62,63,70,81^ or to some other forms of cognitive demand, such as Stroop task performance.^74^ However, by contrast, no conditions positively activated the local NPS pattern in the dorsal posterior insula. Therefore, these results provide new information on the boundary conditions for NPS activation; a nonzero NPS value is not sufficient to discriminate pain from dimensions of breathlessness, such as work of breathing, anticipation of breathlessness, and basic sensorimotor activity. These findings agree with recommendations from Wager et al^83^ and Krishnan et al,^46^ who observed some relative variation in NPS responses to nonpainful warmth and observed pain but without inducing activation of the same magnitude as painful stimuli. However, they contrast with a number of previous studies that have not found significant NPS responses during anticipated pain.^46,56^ The findings thus suggest that new classifiers, perhaps tree-based or rule-based classifiers centred on conjunctions of local pattern responses in specific areas, may be required to achieve further specificity. In this regard, the dorsal posterior insula (dpIns) may be a key region because dpIns (and local NPS pattern in this region) is routinely activated during painful stimuli,^28^ but does not seem to respond to any of the challenges studied here.

### 4.2. Specificity of neurologic pain signatures

These results help us to understand and explore the current boundaries of an established NPS. Although global NPS-related activity was significantly activated by nonpain conditions, qualitative pattern differences existed within the regional responses across specific areas. In addition, although we cannot test whether the underlying activity of the thousands of neurons within each voxel is the same (or even similar) using the resolution afforded with noninvasive fMRI,^87^ sensorimotor areas and the bilateral insula demonstrated local voxel-wise activity patterns that statistically matched those trained on painful stimuli. By contrast, the dorsal posterior insula was not positively activated by any of the conditions tested here. The dorsal posterior insula has been frequently implicated as having a critical role in pain perception^14,20,36,40,72,77^ and may be an essential area in differentiating pain from other salient symptoms. Previous work in both animals^20,40^ and humans^72^ has determined a subregion of the dorsal posterior insula to be a cortical representation of afferent nociceptive stimuli, and thus it could be considered as an important primary sensory junction for ascending peripheral pain stimuli. Therefore, it is possible that localized patterns of activity in this specific area of the brain may prove more informative for specific determination of painful from nonpainful stimuli.

### 4.3. Implications for the understanding of breathlessness

Our findings may also provide insight into the somatosensory (and often salient) aspects of breathlessness within the limits of our current experimental model. Current theories regarding the mechanisms and potential treatments for chronic breathlessness often draw heavily on pain models,^48,51,67^ which is understandable considering that they share some phenomenological characteristics. However, with the search for individualised neuromarkers and brain-based treatments for breathlessness becoming an increasing topic of interest,^37,61^ it is imperative to attempt to understand what is specific for breathlessness within brain activity and connectivity patterns, rather than over-rely on models created from other conditions. Here we have demonstrated statistical similarities to the NPS not only with the perception of breathlessness and pain but also even with the anticipation of aversive resistive breathing loads. This suggests that there is a somatosensory component not only within the NPS, but also in the anticipated threat towards the body that each of these stimuli may provide.

### 4.4. Neural signatures of somatomotor signals

Although the brain is believed to contain primary cortices dedicated to specific sensory experiences such as vision, audition, and touch,^29,47,54,64^ processing of sensory signals does not stop at these junctures. We must decode these sensory inputs—together with our expectations of the world around us^7,9,70,73,79^—to determine what they mean for elements of our health and happiness and the potential necessity for any further action. Thus, processing these multiple dimensions of perceptual information requires higher cortical involvement and communications beyond primary sensory cortices. Although multivariate, brain-wide signatures such as the NPS have been developed to specifically determine the pattern of activity associated with perceptions of somatosensory pain,^83,90^ these complex, salient experiences may not be easily discernible from other threatening perceptions or even simply somatomotor signals in some cases.

Here we have shown that not only breathlessness can evoke statistically significant patterns of activity within brain models of pain, but also anticipating breathlessness and even a simple finger opposition task can also significantly activate the NPS. Although the lived experience of these conditions informs us that they are usually easily separable and distinct experiences, they must share common threads in the brain processes they engage. In essence, they all involve the translation of sensory signals to desired motivated behaviours: to avoid the painful stimulus (immediately or in the future), to respond to respiratory compromise, and to conduct finger opposition movements. When we consider the regional NPS responses to these conditions within the brain, we observe statistical significance with breathlessness and finger opposition in the thalamus, secondary sensory cortex, bilateral insula, and dACC. These areas are indeed associated with early sensory processing (thalamus and secondary sensory cortex),^21,65,69^ representations of bodily state (insula),^17–19,76^ and context-specific behaviours towards directed goals (dorsal anterior cingulate)^39^ and thus may provide a representative network of sensation-motivated behaviours. However, as anticipation of breathlessness can also induce significant activity in the NPS, it does not seem that the presence of sensory information flow from the periphery is a necessity to activate this blueprint of somatomotor sensation. Rather, the preparatory, future-oriented expectation of bodily perceptions may be powerful enough to elicit an NPS-related brain response. Notably, many other salient and affective conditions have failed to produce NPS activation in previous studies. One possibility for the discrepancy between these studies and the present ones is that many previous comparison conditions involved emotional responses, which seem to engage substantially different brain systems overall from those engaged by pain. Perhaps finger opposition, counterintuitively, produces activity patterns more similar to the NPS because it engages basic somatic, attentional and action processes without the additional different systems engaged during emotion and cognitive demand.

### 4.5. Caveats and limitations

When interpreting results such as these, which apply a “signature” developed in one cohort to other cohorts tested on different scanners, several caveats must be kept in mind. First, it is unclear from the present results alone whether the magnitude of activation to breathlessness, anticipation, or sensorimotor demand is comparable to that elicited by pain.^83^ The original NPS model used a quantitative “pain/nopain” threshold that depends on the scale of the data, which Wager et al. attempted to equate across studies; however, BOLD signal is nonquantitative in the sense that it provides relative rather than absolute units of activity and is influenced by multiple variables in acquisition and analysis. Equating absolute signal magnitude across studies has not yet been achieved, although this is a goal of “calibrated BOLD” studies. Thus, in an ideal situation, a test of whether breathlessness activates “pain-like” neural systems would include a dose–response curve with multiple levels of stimuli that are known to be painful; the response to breathlessness (etc.) could be measured relative to responses in the NPS (or other measure) on this calibrated, study- (and analysis-) specific scale.

The same issue applies to analyses of task “selectivity” and specificity (a statistical quantity). These are defined in relation to specific comparisons (eg, pain vs breathlessness) with quantitative thresholds for classifying a test observation as one vs the other. This also requires comparing pain to breathlessness and other conditions within the same study and calibrating their relative subjective intensities. Therefore, although we cannot know for sure whether the NPS responses observed here are quantitatively strong enough to be classified as “pain” or that breathlessness evokes NPS responses of a comparable magnitude to pain, we can demonstrate that finding significant responses in the NPS above zero is not a sufficient statistic to assure specificity for pain.

Another caveat that must be considered is that the application of the NPS, or any similar measure, requires the assumption that the test images are aligned with same anatomical space, with similar patterns of signal dropout and artifacts, as the data used for model training. If local regions (eg, relevant parts of the cingulate or other regions) are misaligned for a participant or group, the sensitivity and specificity of the measure will be compromised. Thus far, tests of broad generalizability across cohorts and analysis pipelines have shown good sensitivity and specificity for the NPS,^66,93^ and we did not detect any obvious misalignment here. However, the limitations in specificity we identified might be ameliorated by further standardization of processing choices and pipelines. This standardization could be pursued in parallel to the development and validation of other types of classifier models that do not rely on signals assumed to be linear across a range of nonpainful and painful stimuli.

Finally, it is possible that the use of subjective scores might help us to refine the current limitations and use of the NPS. Here, a limitation of the current studies is that the responses collected after breathlessness stimuli were not consistent, with ratings of “difficulty” and “anxiety” of the breathlessness stimuli in study 1 and “intensity” and “unpleasantness” in study 2, with different anchor descriptions. Although incorporating these scores directly into the analysis may reveal whether the NPS responses related to breathlessness scaled with subjective experience, the presence of the significant NPS responses to both anticipation of breathlessness and finger opposition limits the additional value of these analyses towards the conclusions reported here. However, future work could aim to standardise these subjective measures to more closely represent relevant dimensions of both pain and breathlessness, and this may allow us to better disentangle the parameters of the NPS that are both specific to pain and generalisable across conditions.

## 5. Conclusions and future directions

Hence, what do these results mean for the NPS? And for our understanding of breathlessness? Are we chasing the impossible, where a pattern of whole-brain activity can identify pain and pain alone in an individual? And what would the perception of pain become if the components comprising motivated somatomotor behaviour were removed? We could strive for finer resolutions and better pattern recognition algorithms, with the hope that this specificity exists underneath the noise of functional neuroimaging. Or, with the inherent spatial constraints imposed on us and the diversity of brains among us,^30^ it may be more fruitful to move away from a modular view of the (noninvasively accessible) macroscale brain and consider that the existence of a highly specific “pain activity network” may not be achievable given both the importance of cognitive context in shaping pain and the current functional neuroimaging tools.^2,84^ That is, somatic conditions, such as breathlessness and finger opposition, and even types of anticipatory threat that are sufficiently intense and strongly referred to the body may activate (what has been believed of as) “pain” systems.

Alternatively, we could narrow our initial search to more primary sensory cortices such as the dorsal posterior insula.^14,20,36,40,72,77^ Although the dorsal posterior insula is not believed to be solely specific to pain,^23^ it is likely one of the more specific single regions in the brain, and the NPS local pattern affords greater resolution than simple activity in this area. However, as our findings caution against using the simple criterion of NPS > 0 as a specific indicator of pain, the development of an appropriate quantitative “pain” threshold is still required.^83^ This could be implemented in the form of developing additional initial criteria, such as local NPS activity in areas including the dorsal posterior insula. Furthermore, possibilities also exist to extend these pain signatures into the realms of regional connectivity patterns within dynamic functional networks.^89^ Such extensions may help us towards understanding both brain activity and connectivity, provide clues as to the flow of information between primary sensory cortices and higher cognitive and limbic structures, and offer the required specificity to help develop better biological targets for assessing and treating pain.

## Supplementary Material

SupplementaryMaterialsHarrison2021

## Figures and Tables

**Table 1:** Mean (±SD) physiological variables across conditioned respiratory tasks.

	Unloaded breathing	Anticipation of breathlessness	Breathlessness (inspiratory resistance)

Study 1			
Mouth pressure amplitude (cm H_2_0)	0.35 (0.8)	0.46 (0.9)	14.7 (8.3)*
P_ET_CO_2_ (mm Hg)	35.5 (4.7)	35.1 (5.0)*	35.9 (5.4)*
P_ET_O_2_ (mm Hg)	131.9 (11.8)	131.6 (10.9)	134.4 (13.0)*
Respiratory rate (min^−1^)	11.8 (3.4)	11.5 (3.8)	10.5 (4.5)*

Study 2: Saline			
Mouth pressure amplitude (cmH_2_0)	2.4 (0.5)	3.5 (1.7)*	12.7 (4.1)*
P_ET_CO_2_ (mm Hg)	42.0 (4.5)	41.3 (3.8)	41.3 (4.5)
P_ET_O_2_ (mm Hg)	148.5 (6.0)	149.3 (5.3)	151.5 (6.0)
Respiratory rate (min^−1^)	16.6 (5.2)	15.6 (4.5)	14.6 (5.3)*

Study 2: Remifentanil			
Mouth pressure amplitude (cm H_2_0)	2.0 (0.4)	2.8 (1.0)*	10.9 (3.4)*
P_ET_CO_2_ (mm Hg)	45.8 (4.5)†	45.0 (4.5)†	45.0 (4.5)†
P_ET_O_2_ (mm Hg)	148.5 (7.5)	149.3 (6.8)	152.3 (9.0)
Respiratory rate (min^−1^)	15.9 (4.3)	14.7 (3.9)*	13.6 (4.7)*

* Significantly (*P*< 0.05) different from corresponding unloaded breathing condition,

† Significantly different from corresponding saline condition (applies to remifentanil conditions alone).

P_ET_CO_2_, pressure of end-tidal carbon dioxide; P_ET_CO_2_, pressure of end-tidal oxygen.

**Table 2:** Mean (±SD) subjective ratings across conditioned respiratory tasks.

	Unloaded cue	Breathlessness cue

Study 1		
Difficulty rating	2.8 (3.5)	46.5 (16.0)*
Anxiety rating	2.5 (3.9)	34.0 (18.8)*

Study 2: Saline		
Intensity rating	12 (16)	71 (20)*
Unpleasantness rating	10 (18)	61 (32)*

Study 2: Remifentanil		
Intensity rating	11 (14)	68 (20)*
Unpleasantness rating	7 (11)	49 (26)*,†

* Significantly (*P*< 0.05) different from corresponding unloaded breathing condition,

† Significantly different from corresponding saline condition (applies to remifentanil conditions alone). Data reproduced from previous publications^27,35^

**Table 3:** Neurologic pain signature responses and statistics for the contrasts of interest in each study.

Study	Contrast	NPS response	STD error	T-STAT	*P*	Cohen *d*

1	Anticipation	53.24	10.39	5.12	<0.01	0.81
	Breathlessness	54.62	9.55	5.72	<0.01	0.90
	Finger opposition	70.47	7.72	9.13	<0.01	1.44
2	Anticipation (S)	34.80	11.80	2.95	<0.01	0.68
	Breathlessness (S)	37.81	10.60	3.57	<0.01	0.82
	Anticipation (R)	31.72	6.30	5.04	<0.01	1.16
	Breathlessness (R)	12.84	6.47	1.98	0.06	0.46
	S>R anticipation	−3.01	12.37	−0.24	0.81	−0.06
	S>R breathlessness	18.88	9.30	2.03	0.06	0.47

Study 1 was conducted at 7 T with 40 participants and 14 stimulus repeats, whereas study 2 was conducted at 3 T with 19 participants and 4 stimulus repeats. NPS, neurologic pain signature.
