# Supplementary material for: Investigating the specificity of the neurologic pain signature against breathlessness and finger opposition
Source: Pain. Author manuscript; Available in PMC 2022 Sep 12. (PMC8600542; doi:10.1097/j.pain.0000000000002327)
Supplement: SupplementaryMaterialsHarrison2021 [file NIHMS1734317-supplement-SupplementaryMaterialsHarrison2021.pdf]

Supplementary Material

Investigating the specificity of the Neurologic Pain Signature against breathlessness and finger opposition

Olivia K. Harrison[1,2,3], Anja Hayen[4], Tor D. Wager[5]* and Kyle T. S. Pattinson[2]*

[1] Translational Neuromodeling Unit, Institute of Biomedical Engineering, University of Zurich and ETH Zurich, Zurich, Switzerland
[2] Nuffield Department of Clinical Neurosciences, University of Oxford, Oxford, UK
[3] School of Pharmacy, University of Otago, Dunedin, New Zealand
[4] School of Psychology & Clinical Language Sciences, University of Reading, Reading, UK
[5] USA Department of Psychological and Brain Sciences, Dartmouth College, Hanover, USA
*Authors contributed equally to this work

Key words: pain, breathlessness, inspiratory resistance, interoception, threat

Corresponding author:
Dr Olivia Harrison (née Faull)
School of Pharmacy
University of Otago
Email: olivia.harrison@otago.ac.nz

Supplementary Table 1: *NPS subregion analyses for 7 Tesla data contrasts of interest. Positive regions above the dotted line, negative regions below.*

Anticipation	***NPS***	***Error***	***t statistic***	***p value***	***Cohen's D***
**Vermis**	-0.074	0.133	-0.553	0.583	-0.087
**Right Insula**	6.993	1.813	3.857	<0.001	0.610
**Right primary visual cortex**	-2.435	1.352	-1.801	0.079	-0.285
**Right thalamus**	-0.091	0.263	-3.444	0.732	-0.054
**Left insula**	1.652	0.541	3.056	0.004	0.483
**Right dorsal posterior insula**	-0.527	0.282	-1.868	0.069	-0.295
**Right secondary sensory cortex**	0.817	0.613	1.332	0.191	0.211
**Dorsal anterior cingulate cortex**	2.534	1.599	1.585	0.121	0.251
**Right lateral occipital cortex**	1.717	0.574	2.994	0.005	0.473
**Left lateral occipital cortex**	1.572	0.753	2.087	0.043	0.330
**Right posterior lateral occipital cortex**	1.873	1.512	1.239	0.223	0.200
**Pregenual anterior cingulate cortex**	0.703	0.408	1.723	0.093	0.272
**Left superior temporal sulcus**	0.362	0.702	0.516	0.609	0.082
**Right inferior parietal lobule**	2.696	0.695	3.877	<0.001	0.613
**Posterior cingulate cortex**	0.070	0.433	0.163	0.872	0.026
Breathlessness	*NPS*	*Error*	*t statistic*	*p value*	*Cohen's D*
**Vermis**	0.392	0.125	3.145	0.003	0.497
**Right Insula**	9.087	1.986	4.576	<0.001	0.724
**Right primary visual cortex**	-0.520	1.233	-0.422	0.675	-0.067
**Right thalamus**	0.764	0.217	3.528	0.001	0.558
**Left insula**	2.557	0.530	4.824	<0.001	0.763
**Right dorsal posterior insula**	0.368	0.302	1.218	0.231	0.193
**Right secondary sensory cortex**	2.462	0.657	3.746	0.001	0.592
**Dorsal anterior cingulate cortex**	4.696	1.949	2.409	0.021	0.381
**Right lateral occipital cortex**	0.008	0.565	0.015	0.989	0.002
**Left lateral occipital cortex**	0.209	0.677	0.309	0.759	0.049
**Right posterior lateral occipital cortex**	-0.338	1.470	-0.230	0.819	-0.036
**Pregenual anterior cingulate cortex**	-0.023	0.409	-0.057	0.955	-0.009
**Left superior temporal sulcus**	-1.129	0.642	-1.758	0.087	-0.278
**Right inferior parietal lobule**	1.596	0.603	2.646	0.012	0.418
**Posterior cingulate cortex**	-0.319	0.404	-0.790	0.435	-0.125
Finger opposition	*NPS*	*Error*	*t statistic*	*p value*	*Cohen's D*
**Vermis**	0.804	0.144	5.582	<0.001	0.883
**Right Insula**	13.083	1.464	8.936	<0.001	1.413
**Right primary visual cortex**	9.634	0.727	13.242	<0.001	2.094
**Right thalamus**	0.693	0.146	4.754	<0.001	0.752
**Left insula**	4.063	0.426	9.548	<0.001	1.510
**Right dorsal posterior insula**	0.250	0.226	1.109	0.274	0.175
**Right secondary sensory cortex**	1.406	0.610	2.305	0.027	0.365
**Dorsal anterior cingulate cortex**	10.500	1.377	7.623	<0.001	1.205
**Right lateral occipital cortex**	-1.830	0.536	-3.412	0.002	-0.539
**Left lateral occipital cortex**	-4.492	0.484	-9.275	<0.001	-1.467
**Right posterior lateral occipital cortex**	-3.520	0.711	-4.951	<0.001	-0.783
**Pregenual anterior cingulate cortex**	0.569	0.291	1.956	0.058	0.309
**Left superior temporal sulcus**	-0.894	0.685	-1.305	0.200	-0.206
**Right inferior parietal lobule**	0.722	0.550	1.312	0.197	0.207
**Posterior cingulate cortex**	-0.311	0.282	-1.103	0.277	-0.174

Supplementary Table 2: *NPS subregion analyses for 3 Tesla data contrasts of interest. Positive regions above the dotted line, negative regions below.*

Anticipation (saline)	***NPS***	***Error***	***t statistic***	***p value***	***Cohen's D***
**Vermis**	-0.056	0.206	-0.274	0.788	-0.063
**Right Insula**	3.290	2.638	1.247	0.229	0.286
**Right primary visual cortex**	-3.703	1.575	-2.352	0.031	-0.540
**Right thalamus**	-0.186	0.366	-0.509	0.617	-0.117
**Left insula**	0.681	0.778	0.875	0.394	0.201
**Right dorsal posterior insula**	-1.304	0.452	-2.885	0.010	-0.662
**Right secondary sensory cortex**	0.265	0.806	0.329	0.746	0.075
**Dorsal anterior cingulate cortex**	1.122	3.667	0.306	0.763	0.070
**Right lateral occipital cortex**	0.053	0.593	0.089	0.930	0.020
**Left lateral occipital cortex**	1.502	1.280	1.174	0.257	0.269
**Right posterior lateral occipital cortex**	1.149	1.872	0.614	0.547	0.141
**Pregenual anterior cingulate cortex**	0.336	1.117	0.301	0.767	0.069
**Left superior temporal sulcus**	2.358	1.348	1.749	0.098	0.401
**Right inferior parietal lobule**	0.707	1.006	0.703	0.492	0.161
**Posterior cingulate cortex**	-0.840	0.569	-1.475	0.158	-0.338
Anticipation (remifentanil)	*NPS*	*Error*	*t statistic*	*p value*	*Cohen's D*
**Vermis**	0.269	0.175	1.540	0.141	0.353
**Right Insula**	4.008	1.576	2.544	0.020	0.584
**Right primary visual cortex**	-3.096	1.135	-2.728	0.014	-0.626
**Right thalamus**	0.350	0.211	1.656	0.115	0.380
**Left insula**	1.212	0.729	1.662	0.114	0.381
**Right dorsal posterior insula**	-0.368	0.387	-0.949	0.355	-0.218
**Right secondary sensory cortex**	-0.484	0.715	-0.677	0.507	-0.155
**Dorsal anterior cingulate cortex**	2.013	2.063	0.976	0.342	0.224
**Right lateral occipital cortex**	0.432	0.570	0.758	0.459	0.174
**Left lateral occipital cortex**	2.340	1.323	1.769	0.094	0.406
**Right posterior lateral occipital cortex**	2.891	1.162	2.487	0.023	0.571
**Pregenual anterior cingulate cortex**	-0.630	0.680	-0.926	0.367	-0.213
**Left superior temporal sulcus**	2.036	0.803	2.536	0.021	0.582
**Right inferior parietal lobule**	0.511	0.816	0.626	0.539	0.144
**Posterior cingulate cortex**	-0.108	0.389	-0.278	0.784	-0.064
Saline > Remi Anticipation	*NPS*	*Error*	*t statistic*	*p value*	*Cohen's D*
**Vermis**	-0.326	0.293	-1.111	0.282	-0.255
**Right Insula**	-0.719	3.200	-0.225	0.825	-0.052
**Right primary visual cortex**	-0.607	1.630	-0.373	0.714	-0.085
**Right thalamus**	-0.536	0.426	-1.260	0.225	-0.289
**Left insula**	-0.530	1.205	-0.440	0.666	-0.101
**Right dorsal posterior insula**	-0.936	0.674	-1.389	0.183	-0.319
**Right secondary sensory cortex**	0.749	1.126	0.665	0.515	0.153
**Dorsal anterior cingulate cortex**	-0.891	4.544	-0.196	0.847	-0.045
**Right lateral occipital cortex**	-0.379	0.893	-0.425	0.676	-0.098
**Left lateral occipital cortex**	-0.838	1.590	-0.527	0.605	-0.121
**Right posterior lateral occipital cortex**	-1.742	1.976	-0.882	0.390	-0.202
**Pregenual anterior cingulate cortex**	0.966	1.477	0.654	0.522	0.150
**Left superior temporal sulcus**	0.322	1.566	0.206	0.840	0.047

**Right inferior parietal lobule**	0.196	0.882	0.222	0.827	0.051
**Posterior cingulate cortex**	-0.732	0.663	-1.104	0.285	-0.253
Breathlessness (saline)	*NPS*	*Error*	*t statistic*	*p value*	*Cohen's D*
**Vermis**	0.089	0.088	1.018	0.323	0.234
**Right Insula**	5.807	1.285	4.519	<0.001	1.037
**Right primary visual cortex**	0.821	1.043	0.787	0.442	0.181
**Right thalamus**	0.475	0.101	4.684	<0.001	1.075
**Left insula**	1.497	0.368	4.066	<0.001	0.933
**Right dorsal posterior insula**	0.018	0.150	0.122	0.904	0.028
**Right secondary sensory cortex**	1.667	0.360	4.636	<0.001	1.064
**Dorsal anterior cingulate cortex**	3.424	1.350	2.537	0.021	0.582
**Right lateral occipital cortex**	-0.603	0.573	-1.052	0.308	-0.241
**Left lateral occipital cortex**	-0.700	0.612	-1.145	0.268	-0.263
**Right posterior lateral occipital cortex**	0.536	0.962	0.558	0.584	0.128
**Pregenual anterior cingulate cortex**	0.882	0.271	3.254	0.005	0.746
**Left superior temporal sulcus**	-0.595	0.583	-1.021	0.322	-0.234
**Right inferior parietal lobule**	0.166	0.409	0.406	0.690	0.093
**Posterior cingulate cortex**	0.035	0.290	0.122	0.904	0.028
Breathlessness (remifentanil)	*NPS*	*Error*	*t statistic*	*p value*	*Cohen's D*
**Vermis**	0.005	0.102	0.050	0.961	0.011
**Right Insula**	0.993	1.196	0.831	0.417	0.191
**Right primary visual cortex**	0.409	0.970	0.422	0.678	0.097
**Right thalamus**	0.026	0.095	0.269	0.791	0.062
**Left insula**	-0.008	0.317	-0.024	0.981	-0.006
**Right dorsal posterior insula**	-0.453	0.174	-2.613	0.018	-0.599
**Right secondary sensory cortex**	0.521	0.455	1.145	0.267	0.263
**Dorsal anterior cingulate cortex**	0.245	1.253	0.195	0.847	0.045
**Right lateral occipital cortex**	-0.193	0.395	-0.489	0.631	-0.112
**Left lateral occipital cortex**	-0.668	0.598	-1.118	0.278	-0.256
**Right posterior lateral occipital cortex**	1.306	0.915	1.428	0.171	0.328
**Pregenual anterior cingulate cortex**	0.756	0.246	3.075	0.007	0.705
**Left superior temporal sulcus**	0.281	0.578	0.486	0.633	0.112
**Right inferior parietal lobule**	0.444	0.443	1.002	0.330	0.230
**Posterior cingulate cortex**	0.236	0.237	0.996	0.332	0.227
Saline > Remi breathlessness	*NPS*	*Error*	*t statistic*	*p value*	*Cohen's D*
**Vermis**	0.084	0.116	0.724	0.479	0.166
**Right Insula**	4.813	1.731	2.781	0.013	0.638
**Right primary visual cortex**	0.412	1.107	0.372	0.714	0.085
**Right thalamus**	0.449	0.142	3.164	0.006	0.726
**Left insula**	1.505	0.494	3.049	0.007	0.699
**Right dorsal posterior insula**	0.472	0.165	2.854	0.011	0.655
**Right secondary sensory cortex**	1.146	0.431	2.657	0.017	0.610
**Dorsal anterior cingulate cortex**	3.179	1.640	0.938	0.069	0.445
**Right lateral occipital cortex**	-0.409	0.430	-0.953	0.354	-0.219
**Left lateral occipital cortex**	-0.032	0.647	-0.050	0.961	-0.011
**Right posterior lateral occipital cortex**	-0.770	0.870	-0.885	0.388	-0.203
**Pregenual anterior cingulate cortex**	0.126	0.376	0.336	0.741	0.077
**Left superior temporal sulcus**	-0.876	0.642	-1.363	0.191	-0.313
**Right inferior parietal lobule**	-0.277	0.426	-0.651	0.524	-0.149
**Posterior cingulate cortex**	-0.200	0.296	-0.676	0.508	-0.155

Supplementary Figures

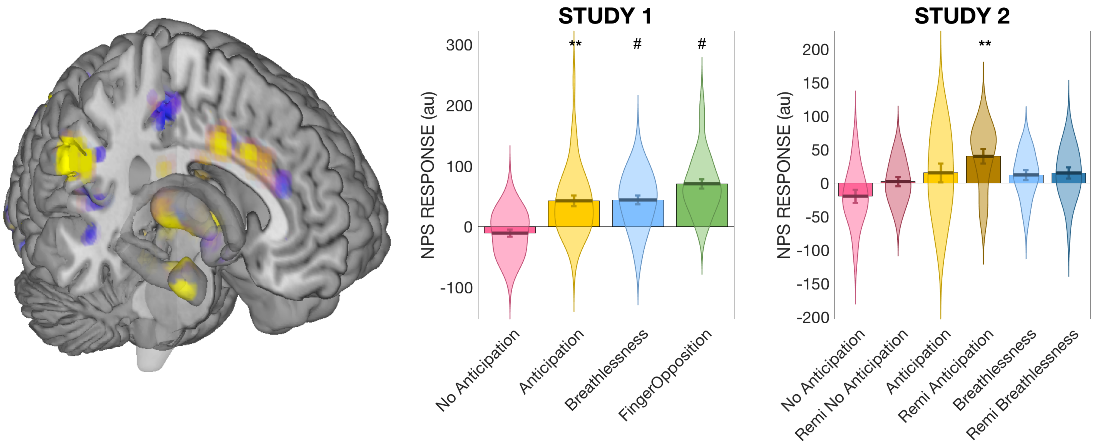

Supplementary Figure 1. Overall NPS activity in all conditions for the two datasets. Left: Three-dimensional representation of some of the core regions of the NPS. ** Significantly different from zero at $p < 0.01$; # Significantly different from zero at $q < 0.05$ (FDR corrected).

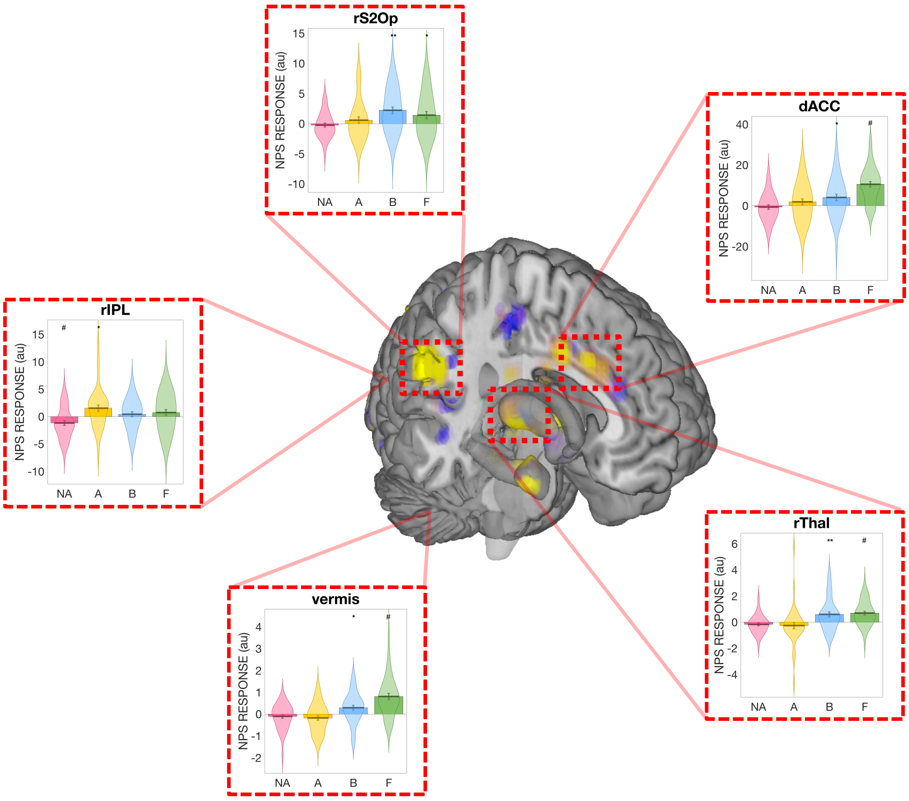

Supplementary Figure 2. Regional NPS activity subregions of the NPS for the no-anticipation, anticipation, breathlessness and finger opposition conditions from Study 1. Abbreviations: dACC, dorsal anterior cingulate cortex; rThal, right thalamus; rS2Op, right secondary somatosensory cortex / operculum; rIPL, right inferior parietal lobule; NA, No anticipation; A, Anticipation; B, Breathlessness; F, Finger opposition. * Significantly different from zero at $p < 0.05$; ** Significantly different from zero at $p < 0.01$; # Significantly different from zero at $q < 0.05$ (FDR corrected).

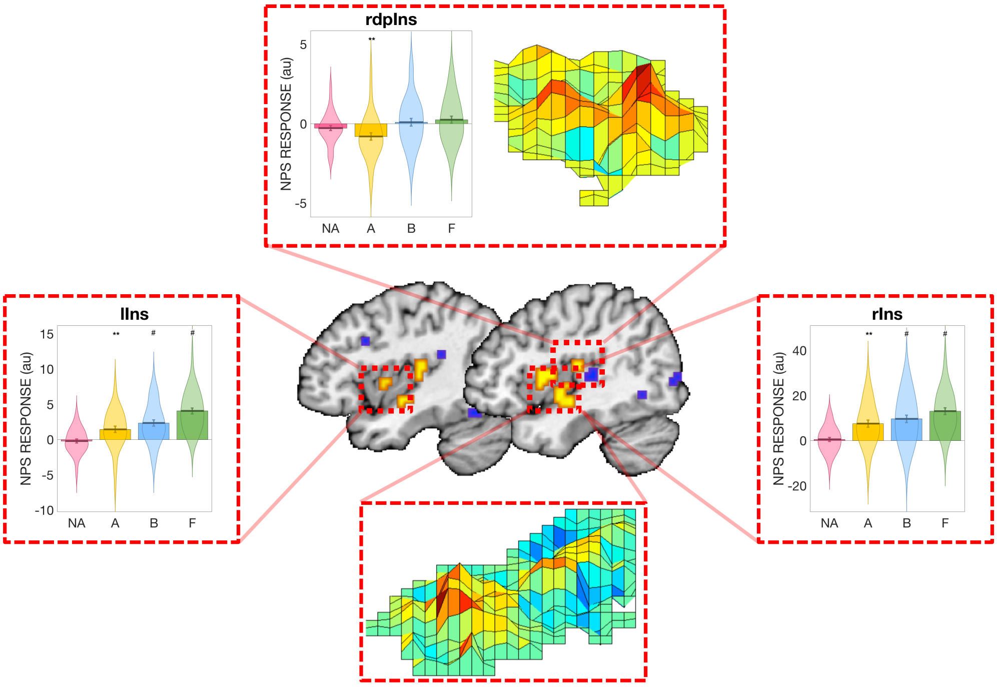

Supplementary Figure 3. Regional NPS activity in the insula for the no-anticipation, anticipation, breathlessness and finger opposition conditions from Study 1. Robust statistical activity is observed in the bilateral insula (labelled lIns and rIns) for all except the no-anticipation condition, while no significant positive activity is observed in the right dorsal posterior insula (rdpIns). Abbreviations: NA, No anticipation; A, Anticipation; B, Breathlessness; F, Finger opposition. ** Significantly different from zero at $p < 0.01$; # Significantly different from zero at $q < 0.05$ (FDR corrected).

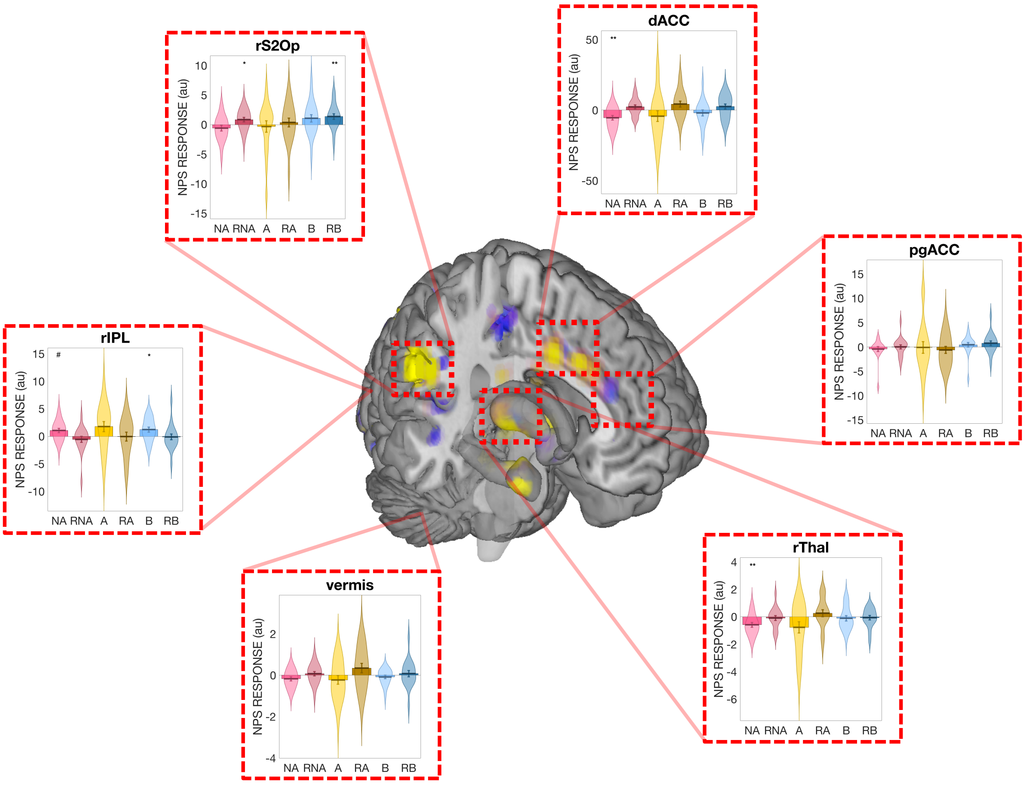

Supplementary Figure 4. Regional NPS activity subregions of the NPS for the no-anticipation, anticipation and breathlessness conditions during both saline and remifentanil administration from Study 2. Abbreviations: dACC, dorsal anterior cingulate cortex; pgACC, pregenual anterior cingulate cortex; rThal right thalamus; rS2Op, right secondary somatosensory cortex / operculum; rIPL, right inferior parietal lobule; A, Anticipation contrast (saline); RA, Remifentanil anticipation contrast; B, Breathlessness contrast (saline); RB, Remifentanil breathlessness contrast. * Significantly different from zero at $p < 0.05$; ** Significantly different from zero at $p < 0.01$; # Significantly different from zero at $q < 0.05$ (FDR corrected).

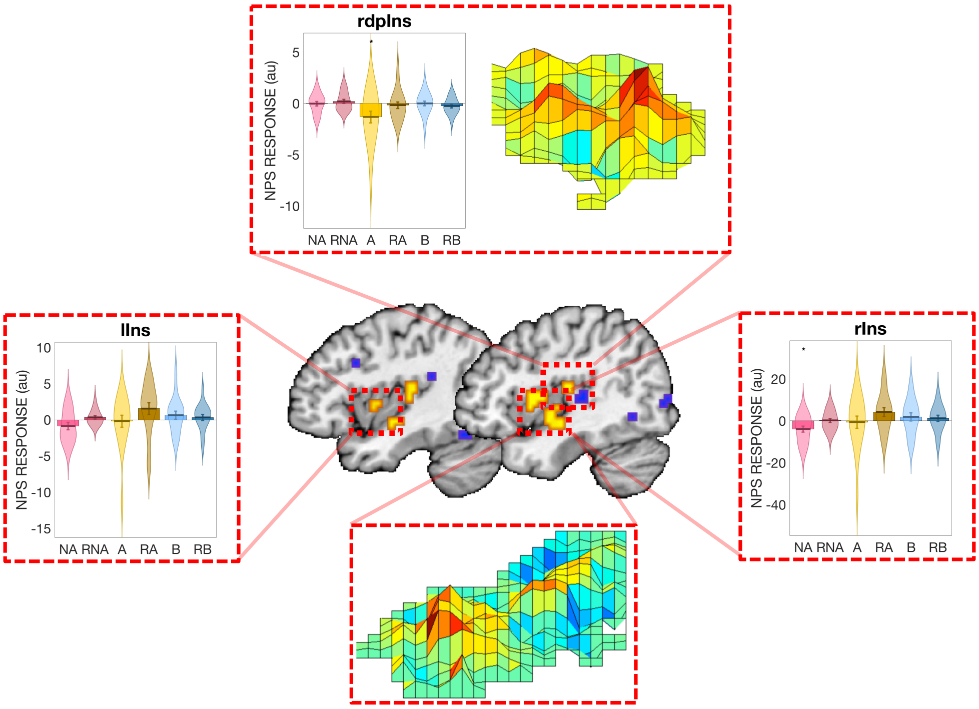

Supplementary Figure 5. Regional NPS activity in the insula for the no-anticipation, anticipation and breathlessness conditions during both saline and remifentanil administration from Study 2. Abbreviations: rIns, right insula; lIns, left insula; rdpIns, right dorsal posterior insula; NA, No anticipation; A, Anticipation (saline); RA, Remifentanil anticipation; B, Breathlessness (saline); RB, Remifentanil breathlessness. ** Significantly different from zero at $p < 0.01$; # Significantly different from zero at $q < 0.05$ (FDR corrected).
